# Changes in Metamorphopsia, Visual Acuity, and Central Macular Thickness after Epiretinal Membrane Surgery in Four Preoperative Stages Classified with OCT B-Scan Images

**DOI:** 10.1155/2019/7931654

**Published:** 2019-06-17

**Authors:** Jianbo Mao, Hanfei Wu, Chenyi Liu, Chenting Zhu, Jimeng Lao, Yiqi Chen, Jiwei Tao, Yun Zhang, Lijun Shen

**Affiliations:** ^1^Department of Retina Center, Affiliated Eye Hospital of Wenzhou Medical University, Hangzhou, Zhejiang Province, China; ^2^The People's Hospital of Zhuji, Zhuji, Zhejiang Province, China; ^3^Chicago College of Optometry, Midwestern University, Downers Grove, IL, USA; ^4^The Second Hospital of Jiaxing, Jiaxing, Zhejiang Province, China

## Abstract

**Purpose:**

To observe the changes in metamorphopsia, visual acuity, and central macular thickness (CMT) in patients undergoing vitrectomy for idiopathic epiretinal membranes (iERM); all of which were preoperatively stratified into 4 stages according to the anatomical structure of the macula seen on the optical coherence tomography (OCT) b-scan images.

**Methods:**

A total of 108 eyes of 106 patients were included. We evaluated and classified the severity of each preoperative ERM based on OCT. Changes in the best-corrected visual acuity (BCVA), metamorphopsia, and CMT were studied by comparing the pre- and postoperative measurements. The follow-up time was at least 6 months.

**Results:**

There were 41 eyes at stage 2, 35 at stage 3, 32 at stage 4, and none at stage 1. BCVA and metamorphopsia significantly improved at the final visit in all patients (*P* < 0.01). However, comparing the pre- and postoperative measurements at each stage, only the BCVA and CMT improved significantly for all stages (*P* < 0.001). For stages 2 and 3 ERMs, the horizontal (MH) and vertical (MV) metamorphopsia scores decreased significantly after surgery (*P* < 0.05). No significant difference was found in either MH or MV for stage 4 ERMs (*P* both >0.05). The preoperative BCVA, MH, and CMT had significant difference among the three stages (*P* < 0.05). Similarly, the postoperative values in the three variables mentioned above also had significant difference among the three stages (*P* < 0.05). For stage 2 ERMs, the baseline MH and MV were positively correlated with the baseline CMT. The MH and MV at the final follow-up also presented a significant positive correlation with the baseline CMT. For stage 3 ERMs, only the baseline MV showed significant correlation with the CMT.

**Conclusion:**

Categorization of the preoperative ERMs is a useful method to predict the postoperative improvement in metamorphopsia, which would aid in surgical decisions for patients with ERMs.

## 1. Introduction

Idiopathic epiretinal membrane (iERM) is a common retinal disorder with an occurrence of approximately 5.3% to 18.5% in the general population [1–5].

Its pathologic feature is characterized by the fibrocellular proliferation of the internal limiting membrane at the macula area, resulting in disturbance of visual function [6]. There are usually no symptoms at early stage of epiretinal membrane (ERM), whereas advanced ERM will likely cause various degrees of visual impairments. Reduced visual acuity and metamorphopsia are the most significant symptoms in patients with iERM and may affect quality of life.

Many researchers have studied the changes of foveal microstructure in ERM, including the integrity of ellipsoid zone, central macular thickness (CMT), and photoreceptor outer segments [7, 8], to identify the anatomic changes that may affect the prognosis after surgery [9–12]. Despite a successful membrane removal and an improvement in the visual acuity (VA), the quality of vision may not be completely enhanced mainly because of the residual metamorphopsia. Kinoshita et al. [13] found that the prognosis for improvement in the metamorphopsia after surgery was strongly related to the severity of the preoperative metamorphopsia. Bae et al. [14] also found that the reduction of the metamorphopsia paralleled the improvement of the BCVA and CMT after iERM surgery. The severity of preoperative metamorphopsia and CMT and integrity of the photoreceptor inner segment/outer segment (IS/OS) junction at the baseline were the significant predictors for postoperative outcome in attenuation of metamorphopsia. All the above indicated that the iERM removal should be performed before development of any severe metamorphopsia. Okamoto et al., otherwise, proposed that the inner nuclear layer (INL) thickness might be a predictor for the postoperative metamorphopsia in patients with iERM [15, 16].

Several classifications for iERM exist according to different standards [17–20]. Only a few of them classified iERM based on a clinical scale or optical coherence tomography (OCT) finding [21]. Rarely has preoperative classification been used as a predictor for the prognosis of postoperative metamorphopsia [22, 23]. Therefore, our study aimed to explore a system of categorization for preoperative iERM to predict the postoperative visual function. Given the known correlation between vision loss and changes in the inner retinal layers in patients with iERM [15, 16, 24–26], the presence of continuous ectopic inner foveal layers on OCT images was chosen as a reference to classify various preoperative iERMs.

## 2. Methods

This was a retrospective study that enrolled one-hundred and six consecutive patients with iERM, who underwent vitrectomy and membrane peel at the Affiliated Eye Hospital of Wenzhou Medical University (Hangzhou, China) from January 2014 to April 2017. This study conformed to the tenets of the Declaration of Helsinki and was approved by the Ethics Committee of the Affiliated Eye Hospital of Wenzhou Medical University.

ERM was defined as a thin membrane attached to the surface of the retina, with or without causing distortion and could be detected on the OCT. Exclusion criteria included history of vitreoretinal surgery, photocoagulation, retinal vascular diseases, uveitis, trauma, and secondary macular membranes such as age-related macular degeneration and any follow-up sooner than 6 months after the membrane removal. Patients with a preoperative BCVA (logMAR) of worse than 1.0 logMAR were also excluded due to difficulty distinguishing change in the metamorphopsia [27].

**Figure 1:**
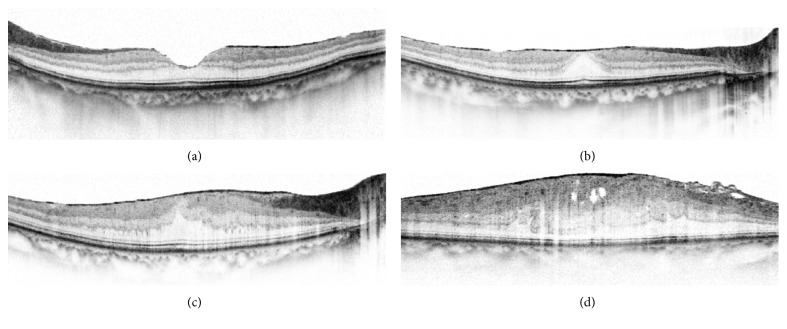
(a) Stage 1: (a) presence of the foveal pit with minimal epiretinal membrane; (b) distinguishable retinal layers. (b) STAGE 2: (a) absence of foveal pit; (b) distinguishable retinal layers. (c) STAGE 3: (a) presence of ectopic inner foveal layer; (b) distinguishable retinal layers despite some distortion. (d) STAGE 4: (a) presence of ectopic inner foveal layer; (b) undistinguishable retinal layers due to severe distortion.

Continuous ectopic inner foveal layers, by definition, is the appearance of a continuous hypo- or hyperreflective band on OCT, through the inner nuclear layer (INL) and inner plexiform layer (IPL) at the fovea [28] (Figure 1(c)).

VA measurement, metamorphopsia scores, and CMT were obtained preoperatively and at every postoperative follow-up. The initial follow-up must occur no sooner than 6 months after the ERM removal, given that the improvement in metamorphopsia plateaued at 6 months after surgery [27]. The mean follow-up interval was 12.31 ± 9.48 months (range, 6–47 months). Only the preoperative and the last postoperative data were used for analysis. Distant BCVA was measured with the Snellen chart and recorded in logarithm of minimum angle of resolution (LogMAR). Retinal images were obtained with the Spectralis OCT (Heidelberg, Germany) instrument. The distance between the internal limiting membrane (ILM) and the surface of retinal pigment epithelium (RPE) at the fovea was used to record the CMT. Average of three consecutive CMT measurements was used for analysis.

The severity of metamorphopsia was evaluated using the M-CHARTS (Inami, Co.), which consists of 1 solid line and 19 dotted lines. The line intervals range from 0° to 2.0° of visual angle. Vertical solid line (0°) was first shown to the patient. If the patient could recognize a straight line as straight, the metamorphopsia score would be 0. However, if the patient recognized a solid line as curved, the dotted lines would then be shown to the patient until the dotted line was perceived straight. If the patient finally recognized a dotted line as straight at 1.5° of visual angle, then the metamorphopsia score would be recorded as 1.5°. After that, the horizontal lines would be presented and tested as for the vertical lines [15]. The examinations were repeated two times for each direction of lines. The mean scores were used for data analyses.

All patients' ERMs were stratified into 4 preoperative stages based on the OCT B-scan images in light of the standard in Andrea Govetto's study, presented as follows [28]:

- Stage 1: the presence of a mild ERM with negligible anatomic disruption. All retinal layers were clearly identified (Figure 1(a)).
- Stage 2: the presence of ERMs associated with more progressive retinal distortion. The normal foveal contour had disappeared, and the outer nuclear layer (ONL) was stretched. However, all retinal layers could still be recognized on OCT images (Figure 1(b)).
- Stage 3: the presence of continuous ectopic inner foveal crossing the central foveal area. All retinal layers were clearly identified on OCT (Figure 1(c)).
- Stage 4: the presence of significant retinal thickness and continuous ectopic inner foveal layers extending from the INL and IPL crossing the entire foveal area. Retinal layers cannot be clearly identified with OCT (Figure 1(d)).

All procedures were performed by a single surgeon (L. J. Shen) using small-gauge pars plana vitrectomy. During the operation, ERM and internal limiting membrane (ILM) in all patients were completely removed with the aid of indocyanine green (ICG, 0.2 ml of 0.5%). Cataract surgery was performed prior to vitrectomy for patients over age 50 (*n* = 100). Neodymium-YAG was conducted if posterior capsule opacification was present at follow-up.

One-way analysis of variance (ANOVA) was used to determine the significance of differences among different stages. The pre- and postoperative visual function parameters and CMT were compared with paired *t* tests. The association among BCVA, M-scores, and CMT at each follow-up was assessed using Pearson's correlation coefficient tests. A *P* value of <0.05 was considered significant.

## 3. Results

This study included 108 eyes in 106 consecutive patients, including 35 males and 71 females, with an average age of 66.87 ± 7.98 years. Cataract surgery was performed on 100 eyes. Five were pseudophakic prior to the surgery, and three had no cataracts. Vitrectomy was performed with 23-gauge instruments in all patients. The demographic data collected prior to operation are shown in Table 1.

### 3.1. Comparisons between Pre- and Postoperative Measurements in Best-Corrected Visual Acuity, Metamorphopsia, and Optical Coherence Tomography Parameters without OCT Classification

Table 2 shows the postoperative changes in BCVA, metamorphopsia, and OCT parameters after ERM removal. BCVA significantly improved after surgery, compared with the baseline values (*t* = 8.86, *P* < 0.001). Similarly, both the horizontal (MH) and the vertical scores (MV) of M-CHARTS decreased significantly at the last postoperative follow-up (*t* = 4.23, 2.96, *P* < 0.001, <0.001, respectively). In addition, the CMT measurement at the last postoperative follow-up was also significantly thinner than the baseline values (*t* = 8.87, *P* < 0.001).

### 3.2. Comparisons between Pre- and Postoperative Measurements in Best-Corrected Visual Acuity, Metamorphopsia, and Optical Coherence Tomography Parameters under OCT Classification

**Figure 2:**
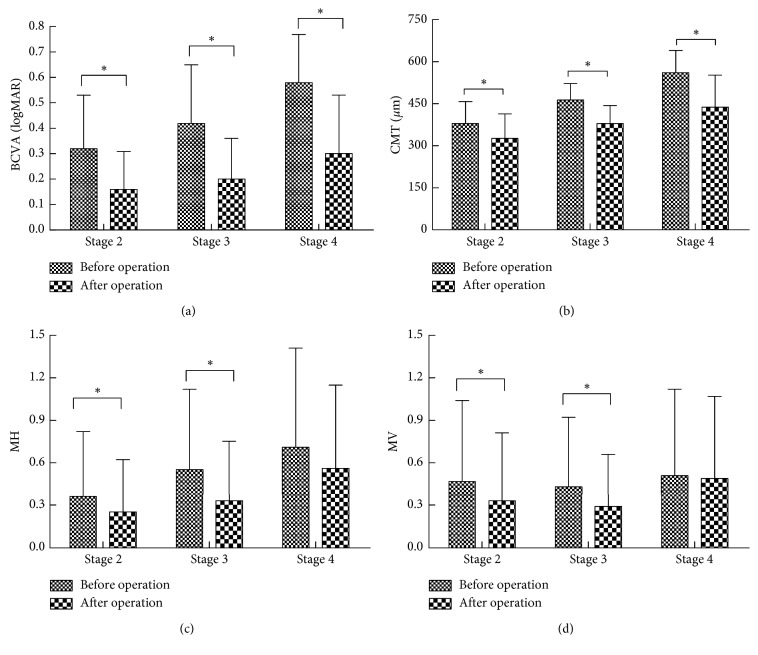
(a) The mean BCVA and different stages before and after surgery to remove ERM. (b) The mean CMT and different stages before and after surgery to remove ERM. (c) The mean MH scores and different stages. (d) The mean MV scores and different stages. Statistically significant compared with preoperation and postoperation by paired *t* test (*P* < 0.05).

At the last follow-up, both the mean BCVA and CMT of patients with stage 2, 3, and 4 iERMs improved from the baseline (*t* = 4.07, 7.06, 5.91; all *P* < 0.001 in BCVA; *t* = 3.64, 7.87, 5.21; all *P* < 0.001 in CMT) (Figures 2(a) and 2(b)). In the meantime, the MH also decreased significantly in patients with stage 2 and 3 iERMs (*t* = 2.34, 3.67; *P* < 0.01). However, there was no significant difference between baseline and postoperative MH in stage 4 iERMs (*t* = 1.51, *P*=0.14). Similar to changes in MH, MV at the final follow-up for stage 2 and 3 iERMs decreased significantly from baseline (*t* = 2.19, 2.40; *P*=0.03, 0.02, respectively), whereas MV at stage 4 did not show significant changes after surgery (*t* = 1.51, *P*=0.14). (Figures 2(c) and 2(d)).

### 3.3. Preoperative Analysis in Best-Corrected Visual Acuity, Metamorphopsia, and Optical Coherence Tomography Parameters under OCT Classification

Statistically significant differences in BCVA were encountered in all 3 subgroups (stages 2, 3, and 4) (*F* = 14.10, *P* < 0.001). The ultimate BCVA in stage 2 iERMs (0.32 ± 0.21) was better than that in stage 3 (0.42 ± 0.23) and 4 (0.58 ± 0.19) (*P* = 0.048, 0.001, respectively), with the ultimate BCVA in stage 3 better than that in stage 4 (*P*=0.002). There was also significantly statistical difference in MH in all subgroups (*F* = 3.35, *P*=0.04). The ultimate MH in stage 2 iERMs (0.36 ± 0.46) was less than that in stage 4 (0.70 ± 0.70) (*P*=0.012). However, no significant difference in MH was found either between stage 2 and stage 3 (*P*=0.148) or between stage 3 and stage 4 (*P*=0.273). MV did not show significant difference among any subgroup (*F* = 0.15, *P*=0.86). Statistically significant differences in CMT were present in all the subgroups (*F* = 56.30, *P* < 0.001). The ultimate CMT in stage 2 iERMs (379.93 ± 77.92) was thinner than that in stage 3 (463.34 ± 59.21) and stage 4 (561.78 ± 78.80) (*P* < 0.001 for both), with the ultimate CMT in stage 3 thinner than that in stage 4 (*P* < 0.001) (Table 3).

### 3.4. Postoperative Changes in Best-Corrected Visual Acuity, Metamorphopsia, and Optical Coherence Tomography Parameters under OCT Classification

There were statistically significant differences in BCVA among the 3 subgroups (*F* = 6.51, *P*=0.002). The ultimate BCVA in stage 2 iERMs (0.16 ± 0.15) was better than that in stage 4 (0.30 ± 0.22) (*P*=0.001), with the ultimate BCVA in stage 3 (0.33 ± 0.42) better than that in stage 4 (*P*=0.01). However, there was no difference between stage 2 and stage 3 iERMs (*P*=0.58). Significantly statistical difference existed in MH among the 3 subgroups (*F* = 4.48, *P*=0.01). The ultimate MH score in stage 2 iERMs was less than that in stage 4 (*P*=0.004). Similarly, the ultimate MH score in stage 3 iERMs was also less than that in stage 4 (*P*=0.04). No difference in MH was found between stage 2 and stage 3. There was no difference in MV among the 3 subgroups (*F* = 1.55, *P*=0.22). Statistically significant differences in CMT were encountered among the 3 subgroups (*F* = 13.88, *P* < 0.001). The ultimate CMT in stage 2 iERMs (326.34 ± 87.67) was thinner than that in stage 3 (379.26 ± 63.88) and stage 4 (438.09 ± 114.20) (*P* < 0.01 for both), with the ultimate CMT in stage 3 iERMs thinner than that in stage 4 (*P* < 0.001) (Table 4).

### 3.5. Correlation between Visual Acuity, Metamorphopsia, and Optical Coherence Tomography Parameters

In stage 2 iERMs, both the baseline MH and MV showed positive correlations with the baseline CMT (*r* = 0.344, 0.357; *P* = 0.028, 0.017, respectively). In addition, the MH and MV at the final follow-up also had significant positive correlations with the baseline CMT (*r* = 0.357, 0.472; *P* = 0.022, 0.002, respectively). Meanwhile, the final MH had a significant positive correlation with the final BCVA (*r* = 0.401, *P*=0.009). In stage 3 iERMs, the baseline MV showed a significant positive correlation with the baseline CMT and the CMT at the last follow-up (*r* = 0.358, 0.340; *P* = 0.035, 0.046). However, in stage 4 iERMs, neither MH or MV showed significant correlation with other parameters (Tables 5–7).

## 4. Discussion

Our results showed that the postoperative mean BCVA and CMT were significantly improved after membrane peel in eyes with iERMs at any preoperative stages. Kim and associates also reported on rapid improvement in VA and CMT during the first 3 months, which stabilized at 12 months after surgery [29]. In addition, metamorphopsia, both MH and MV, improved significantly after surgery in our patients, which agreed to findings in the previous studies [15, 27, 30].

In this study, we adopted Andrea Govetto's new OCT staging scheme to classify the severity of preoperative iERMs. There were significant differences in both the pre- and postoperative metamorphopsia among stages 2, 3, and 4 iERMs. Both the pre- and postoperative MH in stage 2 iERMs were evidently better than those in stage 4 (*P* < 0.01). The postoperative MH in stage 3 iERMs was also better than that in stage 4 (*P* < 0.01). All of the above indicated that patients with stage 2 and 3 iERMs had less metamorphopsia prior to surgery and better prognosis for visual improvement than patients with stage 4. The general improvement of MH was also reported by Takabatake et al. [31] and Kinoshita et al. [27], whose study included mainly patients with stage 2 and stage 3 iERMs. In our study, neither the pre- or postoperative MV showed any difference among the 3 subgroups, which indicated that the severity of iERM had no correlation with MV. Thus, MV score may provide limited indication on the severity of iERM. The potential reason could be related to the directionality of retinal plasticity confined by the running direction of the retinal nerve fiber and the presence of the optic disc [27].

In the past, OCT studies paid more attention to anatomic changes in the foveal microstructure, including disruption at the IS/OS junction and photoreceptor outer segments which were long believed to be the cause of vision loss in patients with iERM [15, 16]. However, more recent attention has been shifted to the study of the inner retinal anatomy, given more changes occurred in the inner layers' integrity due to the tractional stress from the iERM [15–18]. In our study, improvement in the BCVA, metamorphopsia, and CMT after surgery was more significant in patients with stage 2 iERM than those with stage 3 or stage 4 iERM. Given the relative intact and undisturbed outer segments for each stage, we hypothesized that the reason for minimal effect on visual function in early stage iERM may be related to the minimal damage in the inner retinal layers. This may play an important role in predicting overall visual function in patients with iERM, although the pathophysiological mechanism may be speculative. Furthermore, to avoid severe damage in inner retinal layers, early surgery or iERM could lead to better outcomes in improvement of visual function.

Previous studies have reported on the correlation between CMT and metamorphopsia [14, 32]. In our study, the values of CMT in stage 2 iERM had a significant positive correlation with both the MH and MV metamorphopsia scores (i.e., MH and MV). The values of CMT in stage 3 iERM only had a significant positive correlation with the baseline MV scores. No correlation was found in stage 4 iERMs. It is potential that patients with advanced iERM were incapable of recognizing the subtle changes in metamorphopsia due to the severe disruption in the retinal structure.

Some limitations exist in our study. Firstly, subclinical damages in the outer retinal layers were not investigated and could also be related to the severity of metamorphopsia. Secondly, other classifications of iERM proposed by various studies could also be used to study the effect on change in metamorphopsia after ERM removal.

To conclude, more advanced stages of iERMs will have worse metamorphopsia either before or after surgery. Integrity of the inner retinal layers, aside from that of the outer retinal layers, may also be a useful indication on the severity of metamorphopsia and the prognosis for recovery of the visual function after surgery. Significant improvement in metamorphopsia mainly occurred in stage 2 iERM after surgery. ERM removal at early stage could result in less metamorphopsia postoperatively.

## Figures and Tables

**Table 1:** Baseline demographic data of 108 eyes in 106 patients with iERM.

Parameters	
Sex (male/female)	35/71
Age (*y*)	66.87 ± 7.98
Preoperative stage of ERM, no. (%)	
Stage 1	0 (0.0%)
Stage 2	41 (38.0%)
Stage 3	35 (32.4%)
Stage 4	32 (29.6%)
Preoperative BCVA (logMAR)	0.43 ± 0.23
Preoperative CMT (*μ*m)	460.84 ± 103.64
Preoperative MH	0.53 ± 0.59
Preoperative MV	0.47 ± 0.55
The follow-up interval (month)	11.10 ± 10.35

BCVA, best-correct visual acuity; CMT, central macular thickness; MH, horizontal score of M-CHARTS; MV, vertical score of M-CHARTS.

**Table 2:** Postoperative changes in BCVA, metamorphopsia, and OCT parameters after ERM removal.

Parameters	Before operation	After operation	*t*	*P*
BCVA (logMAR)	0.43 ± 0.24	0.22 ± 0.19	8.86	<0.001^*∗*^
MH	0.53 ± 0.59	0.37 ± 0.47	4.23	<0.001^*∗*^
MV	0.47 ± 0.55	0.36 ± 0.49	2.96	0.004^*∗*^
CMT (*μ*m)	460.84 ± 103.64	378.33 ± 98.99	8.87	<0.001^*∗*^

Values are presented as mean ± standard error. ^*∗*^Paired *t* test.

**Table 3:** Preoperative analysis in best-corrected visual acuity, metamorphopsia, and central macular thickness under OCT classification.

Parameters	Stage 2	Stage 3	Stage 4	*F*	*P*
BCVA (logMAR)	0.32 ± 0.21	0.42 ± 0.23	0.58 ± 0.19	14.10	<0.001^*∗*^
MH	0.36 ± 0.46	0.55 ± 0.57	0.70 ± 0.70	3.35	0.04^*∗*^
MV	0.47 ± 0.57	0.43 ± 0.82	0.55 ± 0.11	0.15	0.86
CMT (*μ*m)	379.93 ± 77.92	463.34 ± 59.21	561.78 ± 78.80	56.30	<0.001^*∗*^

Values are presented as mean ± standard error. ^*∗*^Paired *t* test.

**Table 4:** Postoperative changes in best-corrected visual acuity, metamorphopsia, and central macular thickness under OCT classification.

Parameters	Stage 2	Stage 3	Stage 4	*F*	*P*
BCVA (logMAR)	0.16 ± 0.15	0.18 ± 0.14	0.30 ± 0.22	6.51	0.002^*∗*^
MH	0.25 ± 0.36	0.33 ± 0.42	0.56 ± 0.59	4.48	0.01^*∗*^
MV	0.33 ± 0.48	0.29 ± 0.63	0.49 ± 0.10	1.55	0.22
CMT (*μ*m)	326.34 ± 87.67	379.26 ± 63.88	438.09 ± 114.20	13.88	<0.001^*∗*^

Values are presented as mean ± standard error. ^*∗*^Paired *t* test.

**Table 5:** Correlation between visual acuity, metamorphopsia, and optical coherence tomography parameters in stage 2.

	Baseline BCVA		BCVA at final follow-up		Baseline CMT		CMT at final follow-up	
	*r*	*P*	*r*	*P*	*r*	*P*	*r*	*P*
Baseline MH	0.123	0.444	0.203	0.203	0.344	0.028^*∗*^	−0.142	0.374
MH at final follow-up	0.098	0.541	0.401	0.009^*∗*^	0.371	0.017^*∗*^	0.001	0.994
Baseline MV	0.004	0.979	0.122	0.449	0.357	0.022^*∗*^	−0.139	0.386
MV at final follow-up	−0.029	0.855	0.029	0.859	0.472	0.002^*∗*^	0.201	0.208

**Table 6:** Correlation between visual acuity, metamorphopsia, and optical coherence tomography parameters in stage 3.

	Baseline BCVA		BCVA at final follow-up		Baseline CMT		CMT at final follow-up	
	*r*	*P*	*r*	*P*	*r*	*P*	*r*	*P*
Baseline MH	0.129	0.460	0.257	0.137	0.167	0.338	0.165	0.342
MH at final follow-up	0.003	0.985	0.275	0.110	0.141	0.420	0.182	0.294
Baseline MV	0.175	0.316	0.082	0.641	0.358	0.035^*∗*^	0.340	0.046^*∗*^
MV at final follow-up	0.168	0.334	0.045	0.798	0.296	0.084	0.229	0.186

**Table 7:** Correlation between visual acuity, metamorphopsia, and optical coherence tomography parameters in stage 4.

	Baseline BCVA		BCVA at final follow-up		Baseline CMT		CMT at final follow-up	
	*r*	*P*	*r*	*P*	*r*	*P*	*r*	*P*
Baseline MH	0.066	0.721	−0.106	0.563	0.131	0.476	−0.335	0.061
MH at final follow-up	0.265	0.143	−0.219	0.227	0.242	0.183	−0.267	0.140
Baseline MV	−0.192	0.294	0.032	0.861	0.079	0.667	0.013	0.945
MV at final follow-up	−0.149	0.414	−0.186	0.307	0.297	0.099	0.066	0.719

## Data Availability

The data used to support the findings of this study are available from the corresponding author upon request.
